# Frequency of Breakfast Eating and Obesity Prevalence in Primary School Teachers

**DOI:** 10.3390/ijerph19095331

**Published:** 2022-04-27

**Authors:** Martina Uvacsek, Georgina Simkó, Judit Boda-Ujlaky, Zsuzsanna Kneffel

**Affiliations:** 1 Department of Health Sciences and Sport Medicine, Hungarian University of Sports Science, 1123 Budapest, Hungary; uvacsek.martina@tf.hu (M.U.); simko.georgina@tf.hu (G.S.); 2 Department of Psychology and Sport Psychology, Hungarian University of Sports Science, 1123 Budapest, Hungary; ujlaky.judit@tf.hu

**Keywords:** health behaviour, school-teachers, breakfast eating, obesity, occupational health, lifestyle

## Abstract

There is a lack of research using objective measures about teachers’ physical characteristics and lifestyle. The purpose of the study was to evaluate the relationship between frequency of breakfast consumption and body size, body composition, blood pressure and lifestyle factors in teachers from Hungary. The study analyzed data collected from 99 female primary school teachers (50.6 ± 6.6 year) before the COVID-19 pandemic. Anthropometric and resting blood pressure measurements were taken for each participant. Questionnaires were used to assess lifestyle factors (i.e., physical activity level, smoking status and eating breakfast). The frequency of eating breakfast was classified as rarely or never (0–2 days), some days (3–5 days) and regularly (6–7 days). Sixty-five percent of female teachers consumed breakfast 6–7 days/week, and this regular eating habit was positively associated with a lower risk of obesity (OR 0.29; CI: 0.10–0.80). In our sample, a higher frequency of breakfast consumption was not significantly associated with smoking (OR 2.65; CI: 0.17–1.37), hypertension (OR 0.61; CI: 0.23–1.65) and inactivity (OR 2.80; CI: 0.26–1.84). A beneficial effect of eating breakfast regularly was found on body composition in female teachers. Further studies should focus on the health behaviors of teachers and their relationship with physical activity or diagnosed diseases in urban and rural areas.

## 1. Introduction

Hungary has the highest obesity rate in Europe and has the fourth-highest prevalence of obesity in the world. According to the Organization for Economic Co-operation and Development (OECD), 30% of Hungarians 15 years of age or older are obese, and 55.5% are obese or overweight [1]. The cause of obesity is a complex interaction between environmental, genetic, behavioral and socioeconomic factors. It is generally accepted that diet and physical activity are modifiable risk factors that are strongly related to being overweight and obesity. Obese people are less productive at work due to more sick days and fewer worked hours, and they earn about 10% less than non-obese people. It is generally true that education and socio-economic background affect the prevalence of obesity, and in the majority of countries, more women are obese than men [1]. According to the Eurobarometer’s latest report, 59% of respondents in Hungary engage in sport or physical activity at home and 29% on the way between home and work or shops [2].

There is a lack of national policies or action plans to promote physical activity in transport or in the environment and at workplaces. The national recommendations were implemented in 2011 based on the WHO’s global guideline on Physical Activity for Health, as well as the recommendations from the US Centers for Disease and Prevention. The target groups of national actions were children and adolescents, but the most concerned population of working adults have not been reached until now [3]. Helo et al. recently reported that people who rarely consume breakfast have a higher risk of cancer-related mortality than those who eat breakfast everyday [4]. Regular breakfast consumption is an indicator of a health-promoting lifestyle, and those who do not eat breakfast are more likely to adopt or continue other unhealthy lifestyle habits, such as smoking, higher alcohol and added sugar consumption and lower levels of physical activity [5]. In Hungary, about 77,000 people work as teachers in primary schools. In this large academic occupational group, more than 80% are female. Generally, the teaching profession is characterized as stressful work with a high workload, low job satisfaction, low income, poor general well-being and physical health and early retirement [6]. According to OECD data, Hungarian primary-school teachers earn the second-lowest salary in the European Union [7]. Combined, these factors negatively affect overall well-being and physical and mental health and can increase the risk of cardiovascular and metabolic diseases. Teachers’ health status is often assessed using questionnaires, and as a result, there is a lack of research using objective measures of body composition and blood pressure and information about their lifestyle. To the best of our knowledge, this is the first study of Hungarian primary school teachers that combines questionnaire data and anthropometric data.

The objective of the study was to characterize teachers’ health status and compare body sizes, body composition, blood pressure and lifestyle factors of teachers according to the frequency of breakfast consumption.

## 2. Material and Methods

### 2.1. Participants

For the study design, the targeted sample size was 100 participants. According to the Hungarian Statistical Office, the number of primary schools is 401 in Budapest, where about 8400 teachers work full time, with most of them being female (82%). The targeted sample size represents 1% of this special population. 

In this study, we included full-time female teachers over the age of 35 (mean age: 50.6 ± 6.6 year), with a minimum of 10 years of experience working in primary schools in Budapest and Pest County. We excluded administrators and other staff members working in schools. After checking the data, 3 participants were excluded from further analysis because of incomplete data. 

Prior to any data collection, ethical approval was received from the University of Physical Education (TE-KEB/No14/2017), and written informed consent was obtained from each participant in the spirit of the Declaration of Helsinki. All data collection was performed between 9:00 and 11:00 in the morning. Participants were asked not to do any physical activity prior to participating in this study.

### 2.2. Procedures

Anthropometric data were recorded prior to measuring resting blood pressure. Stature and body mass were measured using standard anthropometric techniques with the participants wearing light clothing without shoes. Stature (to the nearest 0.1 cm; Sieber-Hagen, Switzerland) and body mass (to the nearest 0.1 kg; Beuer BG22, Waidhofen, Germany) were recorded according to the ISAK criteria [8]. Body mass index (BMI) was calculated as body mass (kg)/stature (m^2^). Based on the WHO cut-off points, participants with a BMI between 25 and 30 were classified as overweight, and those with a BMI with over 30 were classified as obese [9]. The percentage of body fat (%BF) was estimated from 7 skinfold measurements with a Harpenden skinfold caliper (CE0120, Weston-super-Mare, United Kingdom). The measurements were taken by the same skilled anthropometrist. Following the anthropometric measurements, blood pressure was measured using a fully automated device (OMRON M3 comfort, OMRON Healthcare Europe B.V., Hoofddorp, Netherlands), following standard procedures (5-min rest in a seated position, with the back supported and the arm resting on a table at the level of the heart). Hypertension was defined according to the Hungarian Society of Hypertension as a blood pressure (BP) ≥ 140/90 mmHg [10].

We used a simple questionnaire, which included questions about age, smoking, physical activity and eating breakfast. Because of the time limitations in which we had contact with the teachers during the day, this study only asked essential questions relevant to the research question. All participants were asked “How many days a week do you eat breakfast?” The frequency of breakfast eating was classified as “rarely or never (0–2 days)”, “some days (3–5 days)” and “regularly (6–7 days)”. Participants were also categorized as a smoker or non-smoker based on their self-reported current smoking behaviour. 

The physical activity level was assessed with the following question: “Over the past 7 days, on how many days were you physically active for a total of at least 30 min per day? Please add up all the time you spent doing physical activity each day.” The subjects were separated into three groups: recommended level = frequency of moderate to vigorous physical activity (MVPA) was 5 or more days, insufficient physical activity (PA) = frequency of MVPA was 2–4 days and inactive = frequency of MVPA was 1 day or less.

Study design: ethical approval was received at the end of 2017; informed consent and the study was announced online, and the pilot study started in March 2018; data collection (with a snowball research method) and measurements in the field started in May 2018 (anthropometric data and behavioral data on smoking, physical activity and eating breakfast). Data on each participant were collected in one visit, in the morning at the school in which the teachers were employed. After a convenience sample of female teachers was measured from primarily schools in Budapest and Pest County, the statistical analysis started in February 2020.

### 2.3. Statistical Analysis

The variables of interest included age, body height, body mass, body mass index (BMI), body fat percentage (%BF), blood pressure (BP) and questionnaire data. Data were partitioned into three groups based on the frequency of eating breakfast. Descriptive data were calculated for each group and the total sample (Table 1). The data from the three groups were compared with the ANOVA and Scheffe post hoc tests. Frequency tables were used to observe the distribution of lifestyle characteristics and cardiovascular risk factors. A Kruskal–Wallis analysis of variance for non-parametric variables was used for calculating the differences in lifestyle characteristics. The odds ratios (OR) and 95% confidence intervals (CI) were calculated with a binary logistic regression. All statistical analyses were performed using IBM SPSS Statistics (version 27). Tendency as a terminology was accepted at a *p* value range of 0.1–0.05, and the alpha level of significance was set at *p* < 0.05.

## 3. Results

Most of the teachers (65%) ate breakfast 6 or 7 days/week, 16% of the teachers ate breakfast 3 to 5 days a week and 18% rarely or never ate breakfast (Table 1). There were no significant differences in age, body height, body mass (BMI) and %BF and systolic and diastolic blood pressure between the three groups of teachers.

Based on the lifestyle questions, in our sample, most of the female teachers were non-smokers, were insufficiently active, had a normal nutritional status and had healthy blood pressure. The distributions of lifestyle characteristics (smoking status, physical activity level, BMI and hypertension) were not significantly different between the three groups of teachers (Table 2). However, the teachers who rarely or never consumed breakfast tended to have a higher prevalence of smoking and inactivity. Teachers who consumed breakfast regularly were more likely to be non-obese and normotensive. The results of the logistic regression are presented in Table 3. Hypertension odds ratios were higher in teachers who ate breakfast 3–5 days a week or one day or less, but the confidence intervals were wide. We found that regular breakfast consumption (6–7 days/week) had a significant impact on obesity. That means that regular breakfast was found as a protective factor in this study. Teachers who rarely ate breakfast had the highest odds ratio for obesity. Higher odds ratios were also found in relation to smoking (OR 2.65) and inactivity (OR 2.80) in teachers who rarely or never ate breakfast.

## 4. Discussion

In our study, we found that eating breakfast on a minimum of 6 days/week was associated with a lower risk of obesity (OR 0.29; CI: 0.1–0.8). However, the odds ratios between eating breakfast 3–5 day/week and hypertension (OR 1.93; CI: 0.58–6.34) and obesity (OR 2.27; CI: 0.68–7.56) were not significant. However, we found some tendencies: teachers who rarely or never ate breakfast had a higher risk of developing obesity (*p* = 0.09), smoking (*p* = 0.09) and physical inactivity (*p* = 0.06), but due to the wide confidence intervals, these odds ratios were non-significant.

A previous study of health behaviors of male and female Hungarian teachers reported that the frequency of eating breakfast was higher (60% vs. 40%), but the number of days per week that included MVPA was lower, in male teachers (2.7 vs. 3.0). It was also reported that the teachers’ health behavior did not differ by gender [11]. In Hungarian adolescents, skipping breakfast became more prevalent with age, and less than 40% of adolescents ate breakfast regularly on weekdays. Of the 45 countries that were studied, Hungarians were ranked 40th in breakfast consumption [12]. In a cross-sectional study, there were remarkable differences in the prevalence of parents skipping breakfast within European countries, wherein Hungarian parents had one of the highest (43%) skipping ratios [13]. The data reported in the present study, that 65% of teachers regularly ate breakfast, might be a sign of developed health behaviors, which is in line with a German cross-sectional teachers’ health study, where female teachers’ health behavior was more positive than the general population [14]. Moreover, our result corresponds with a study which found that skipping breakfast was more prevalent in the under-50 age group and that regular breakfast frequency increased in older adults [15]. The nutritional status of female teachers in this sample was healthier than in the general population of Hungary, where the prevalence of obese females was 9% higher (25.6%) [16]. The lower obesity rate may be an indication of their healthier lifestyle. 

The overall prevalence of smokers in this study was 19%, but this ranged from 15–33% between the three groups (Table 2). This is higher than that presented in a German teachers’ study, where only 8% of working female teachers smoked [14]. Regular smoking is a major health concern in the Hungarian adult population, in which approximately 26% of females smoke [16]. Our data indicate that smoking is associated with rarely consuming breakfast. However, in our sample, although the frequency of smokers was double among the breakfast skippers compared to regular breakfast eaters, the difference was not significant. This result might be explained by the small number of subjects represented in the three groups.

We found that 25–29% of teachers reported participation in the recommended level of physical activity, which is lower than previously reported in female teachers’ activity data (46%) [17]. Moreover, the physical activity levels of female Hungarian teachers in this study were lower than those of Hungarian females (60.6%) reported in the European Health Interview Survey [16]. The relatively low physical activity reported in this study might be explained by the misunderstanding of the activity question. In general, the physical activity of the Hungarian population is mainly based on their work and housework activity. Activity achieved by transport or leisure time is not common [18]. In addition, participants may not be able to recall their physical activity for the entire previous 7 days.

The relationship between eating breakfast and obesity is uncertain. In observational studies, skipping breakfast has been associated with increased body composition [19,20] and weight gain [21]. Ma et al. (2003) also found that a greater eating frequency was associated with a lower risk of obesity [22], which has also been demonstrated in young people [23]. However, diverse results were published in randomized controlled trials [24,25]. A meta-analysis of 44 studies by Ma and Xu (2018) found that skipping breakfast significantly increased the risk of obesity compared to eating breakfast. In addition, people who skipped breakfast had an increased risk of hypertension, diabetes and dyslipidemia [26].

Our findings concur with the results of studies reporting a relationship between a low frequency of/skipping breakfast and the incidence of obesity. An explanation of our findings is that skipping breakfast may induce changes in appetite and disturb the appetite-related hormones, causing changes in dietary patterns, which might lead to increased food consumption later in the day and reduced insulin sensitivity [27]. However, eating breakfast has a beneficial effect on appetite regulation and also improves the glycemic response at the next meal with increased sensitivity to insulin [28]. In Hungarian primary schools, the working hours start at 07:30, and the lessons start at 8 o’clock in the morning. Both the early waking time and short (10–15 min) breaks between the classes might have a negative effect on breakfast, as well as on their eating habits throughout the day. The irregular meal timing associated with working conditions and personalities in a Japanese study and a Netherlands cohort study found that unhealthy dietary patterns, in both low and highly educated participants, were reported [29,30]. In socioeconomically disadvantaged neighborhoods in Australia, Smith et al. (2013) found that compared with breakfast consumers, women who reported rarely/never eating breakfast tended to have poorer self-rated health, smoke, pay less attention to health, not prioritize their own healthy eating and have less nutrition knowledge, and a lower proportion were trying to control their weight [31]. Thus, a range of intrapersonal and social factors were significantly associated with skipping breakfast in women living in socioeconomically disadvantaged areas, which is in line with our socioeconomically disadvantaged sample of Hungarian female teachers.

Hypertension is one of the leading cardiovascular risks among Hungarians as well as worldwide. Thus, is it essential to see the health impact of the frequency of eating breakfast on cardiovascular risk factors such as hypertension. In the general population, the hypertension prevalence was 30.9% in 2019; in our sample, we found a significantly lower hypertension ratio (21%) [16]. In some earlier studies, authors identified moderate correlations between eating breakfast and the incidence of cardiovascular diseases [32,33]. Others found that skipping breakfast was associated with an increased risk of hypertension [34]. The explanation of these findings could be the results of changed dietary patterns, which could be a triggering factor of cardiometabolic abnormalities that lead to the development of atherosclerosis and hypertension [35]. However, in our study, we did not find a relationship between the frequency of eating breakfast and hypertension. Thus, our findings are quite similar to Rong et al. (2019), who also found no correlation between eating breakfast and hypertension. The ratio of hypertensive versus normotensive participants and the mean age of our sample could explain the results obtained [36]. 

### Limitations of the Study

The present study has several limitations. First, as this study was a descriptive study involving cross-sectional data rather than longitudinal ones, a causal relationship between eating breakfast and hypertension and body composition could not be definitively established. Second, there was no information on the foods and nutrient composition of the breakfast consumed, and beverages consumed were not included. Third, the non-random sample design of our study may have limited the generalizability of our results. Fourth, we did not assess other confounding factors, such as income or marital status. Fifth, the number of participants was limited because of the outbreak of the COVID-19 pandemic.

## 5. Conclusions

Sixty-five percent of female teachers consumed breakfast 6–7 days/week, and this regular eating habit was positively associated with a lower risk of obesity, thus reinforcing the beneficial effect of eating breakfast. Most of the regular breakfast eaters had a normal nutritional status. Regular breakfast consumption was not significantly associated with smoking, hypertension and inactivity in our sample. Based on our results, similar studies are needed to promote regular physical activity and comprehensive health promotion action plans at workplaces. To assess the prevalence of lifestyle-related diseases among teachers, additional studies can focus on the health behaviors of teachers in urban and rural areas. These further studies can evaluate health behaviors in male and female teachers, with additional data collection (i.e. marital and family status, socioeconomic status and full vs. part-time teachers) and with validated lifestyle and physical activity and dietary questionnaires. 

## Figures and Tables

**Table 1:** Age, anthropometric data and blood pressure values of the study population according to frequency of breakfast consumption (mean ± SD).

	All	Frequency of Breakfast Consumption			
		6–7 Days/Week	Some Days	Rarely or Never	
	(*n =* 99)	(*n =* 65)	(*n =* 16)	(*n =* 18)	*p*
Mean Age	50.43 ± 6.59	50.78 ± 7.07	50.22 ± 6.72	50.06 ± 4.99	0.79
Body Height	162.78 ± 6.31	162.65 ± 6.56	161.71 ± 7.01	163.52 ± 4.84	0.79
Body Mass	68.67 ± 11.01	67.88 ± 10.89	71.65 ± 18.40	72.57 ± 12.41	0.35
BMI	25.77 ± 3.97	25.65 ± 3.80	25.86 ± 4.35	27.12 ± 4.53	0.54
BF%	28.59 ± 5.38	28.49 ± 5.47	28.78 ± 6.19	29.57 ± 4.99	0.73
Systolic BP	124.77 ± 12.78	124.57 ± 12.85	129.12 ± 19.13	123.88 ± 8.29	0.80
Diastolic BP	74.73 ± 6.58	74.75 ± 6.58	74.68 ± 8.87	74.83 ± 4.11	0.99

**Table 2:** Distribution of lifestyle characteristics and cardiovascular risk factors of the study population according to the frequency of breakfast consumption.

	All	Frequency of Breakfast Consumption			
		6–7 d/w	Some Days	Rarely or Never	
		(*n =* 65)	(*n =* 16)	(*n =* 18)	*p*
Smoking status (%)					0.22
Non-smoker	81	85	87	67	
Smoker	19	15	13	33	
Physical activity (%)					0.56
Inactive	23	21	13	39	
Insufficient	49	50	62	33	
Recommended level	28	29	25	28	
BMI categories (%)					0.36
<25.0	47	46	50	39	
25.0–29.9	36	42	19	28	
≥30.0	17	12	31	33	
Hypertension (%)					0.51
Hypertensive	21	18	31	22	
Normotensive	79	82	69	78	

**Table 3:** Odds ratios (OR) and 95% confidence intervals (CI) for hypertension, obesity, smoking and physical inactivity based on the frequency of breakfast consumption.

	Frequency of Breakfast Consumption					
	6–7 Days/Week	*p*	Some Days	*p*	Rarely or Never	*p*
Hypertension	0.61 (0.23–1.65)	0.33	1.93 (0.58–6.34)	0.27	1.09 (0.31–3.74)	0.88
Obesity	0.29 (0.10–0.80) *	0.01	2.27 (0.68–7.56)	0.18	2.65 (0.84–8.34)	0.09
Smoking	0.49 (0.17–1.37)	0.17	0.98 (0.25–3.85)	0.97	2.65 (0.84–8.34)	0.09
Inactivity	0.69 (0.26–1.84)	0.46	0.45 (0.09–2.15)	0.31	2.80 (0.93–8.42)	0.06

* *p* < 0.05.

## Data Availability

All the data associated with the current study have been presented in this manuscript.
